# Electrical Phase Modulation Based on Mid‐Infrared Intersubband Polaritonic Metasurfaces

**DOI:** 10.1002/advs.202207520

**Published:** 2023-04-07

**Authors:** Hyeongju Chung, Inyong Hwang, Jaeyeon Yu, Gerhard Boehm, Mikhail A. Belkin, Jongwon Lee

**Affiliations:** ^1^ Department of Electrical Engineering Ulsan National Institute of Science and Technology (UNIST) Ulsan 44919 Republic of Korea; ^2^ Walter Schottky Institute Technical University of Munich 85748 Garching Germany

**Keywords:** intersubband transitions, metasurface, mid‐infrared, multiple quantum well, phase modulation

## Abstract

Electrically reconfigurable metasurfaces that overcome the static limitations in controlling the fundamental properties of scattered light are opening new avenues for functional flat optics. This work proposes and experimentally demonstrates electrically phase‐tunable mid‐infrared metasurfaces based on the polaritonic coupling of Stark‐tunable intersubband transitions in semiconductor heterostructures and electromagnetic modes in plasmonic nanoresonators. In the applied voltage range of −3 to +3 V, the local phase tuning of the light reflects from the metasurface, which enables the electrical control of the polarization state and wavefront of the reflected wave. Electrical beam polarization control, electrical beam diffraction control, and electrical beam steering are experimentally demonstrated as applications for local phase tunability. The proposed electrically tunable metasurfaces can easily tune the operating wavelength and function at relatively low voltages, which will enable various applications in the mid‐infrared region.

## Graphical abstract

Electrically phase‐tunable mid‐infrared metasurface is reported. Based on the polaritonic coupling of Stark‐tunable intersubband transitions in semiconductor heterostructures and electromagnetic modes in plasmonic nanoresonators, local phase tuning of the light reflected from the metasurface is achieved. With the property, electrical beam polarization control, electrical beam diffraction control, and electrical beam steering are experimentally demonstrated.

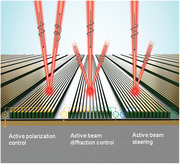

## 1 Introduction

The advent of metasurfaces, namely, engineered surfaces comprising a spatial arrangement of subwavelength unit structures or meta‐atoms, gave rise to a new concept of flat optics owing to their ability to control the amplitude, phase, and polarization of scattered light.^[^ ^1^, ^2^ ^]^ The local controllability of the fundamental properties of light has led to the development of miniaturized optical devices that can replace conventional bulk optics relying on phase accumulation by light propagation. Recently, a vast amount of applied research such as metalens,^[^ ^3^, ^4^, ^5^ ^]^ waveplate,^[^ ^6^, ^7^, ^8^ ^]^ holography,^[^ ^9^, ^10^, ^11^, ^12^ ^]^ and vortex beam generation^[^ ^12^, ^13^, ^14^, ^15^ ^]^ has been reported. However, most of these metasurfaces are passive type optical devices and, once fabricated, their optical properties cannot be changed. To construct multifunctional flat optics to overcome the static limit, attempts have been made to build a reconfigurable metasurface using material property changes caused by mechanical,^[^ ^16^, ^17^, ^18^, ^19^ ^]^ thermal,^[^ ^20^, ^21^, ^22^ ^]^ electrical,^[^ ^23^, ^24^, ^25^, ^26^, ^27^ ^]^ or optical stimuli.^[^ ^28^, ^29^, ^30^ ^]^ Among these methods, electrically tunable metasurfaces, in particular those capable of monolithic integration with other electronics and fast tuning, have received much attention. Wavefront engineering through a reconfigurable metasurface requires independent control of the amplitude and phase with full 360° tuning at the individual meta‐atom level. Recently, carrier concentration control in a transparent conducting oxide layer facilitated an electrical continuous phase sweep between 0° and 360° and its application for light detection and ranging in the near‐infrared region.^[^ ^25^ ^]^ In the mid‐infrared (mid‐IR) region, electrical phase tuning over 230° was demonstrated based on graphene‐gold resonator geometries that require high operational voltages.^[^ ^31^ ^]^ Absorption modulation and fast spectral tunability based on electrically tunable intersubband polaritonic metasurfaces utilizing the strong coupling of optical modes in plasmonic resonators with a giant electro‐optic effect of Stark tunable intersubband transitions (IST) in multiple quantum wells (MQWs) have been demonstrated as another method in the mid‐IR range.^[^ ^27^, ^32^ ^]^ The great advantage of the coupled system lies in its ability to flexibly determine the operating wavelength in the mid‐IR and THz regions by quantum engineering and electromagnetic engineering and produce electrically reconfigurable optical devices with relatively low operational voltages. Such a hybrid structure has also been used to produce electrically tunable nonlinear optical responses.^[^ ^33^ ^]^ However, electrical phase modulation and its applications for polarization control and dynamic wavefront manipulation based on intersubband polaritonic metasurfaces have not been studied yet.

**Figure 1:**
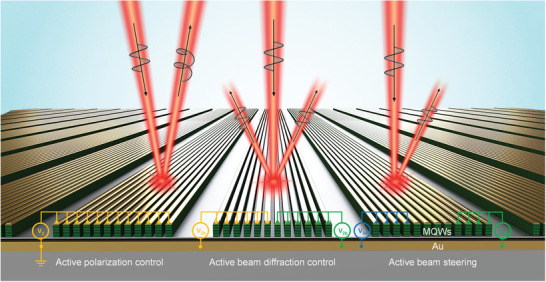
Electrically tunable polaritonic metasurfaces. Schematic illustration of electrically tunable polaritonic metasurfaces and their applications. An multiple quantum well (MQW) layer with giant electro‐optic response is sandwiched between the bottom gold ground plane and top gold layer in double‐metal ridge resonators, in which the local phase of *x*‐polarized beam is controlled by the bias voltage applied. Electrical control of beam polarization state (left), tunable beam diffraction control (middle), and tunable beam steering (right) are demonstrated in a single device through the polarization‐sensitive local phase tuning.

In this study, we propose and experimentally demonstrate electrically tunable intersubband polaritonic metasurfaces in which the local reflection amplitude and phase at the individual meta‐atom level can be controlled by an external bias voltage through the quantum‐confined Stark effect (QCSE) in ISTs.^[^ ^34^, ^35^ ^]^ The local reflection amplitude and phase tuning enables the implementation of a single metasurface with versatile functionalities, such as electrical polarization state control and wavefront tuning. As an example application of the proposed metasurface, we experimentally demonstrate an electrical control of beam polarization state and electrical tuning of beam diffraction and beam steering. A conceptual schematic of the multifunctional operations of our electrically tunable intersubband polaritonic metasurfaces is presented in **Figure** **1**.

## 2 Results and Discussion

**Figure 2:**
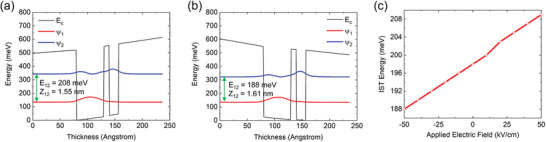
Electrical modulation of the intersubband transition (IST) energy of the multiple quantum well (MQW) structure. a) Conduction band diagram of the In_0.52_Al_0.48_As/In_0.53_Ga_0.47_As coupled quantum well unit structure under a) 50 kV cm^−1^ (+1 V over a 200 nm‐thick MQW) and b) −50 kV cm^−1^ (−1 V) bias field. *E*_12_ and eZ_12_ indicate the IST energy and transition dipole matrix element between the two electron subbands, respectively, where *e* is the electron charge. *E*_c_ represents conduction band edge and *ψ*_1_, *ψ*_2_ represent the first and second electron wavefunction in the conduction band quantum well, respectively. c) Calculated result for the IST energy of *E*_12_ as a function of the applied electric field (or bias voltage) by the quantum‐confined Stark effect (QCSE).

To build an electrically tunable intersubband polaritonic metasurface, we used an n‐doped MQW structure with a giant electro‐optic effect through the QCSE in ISTs.^[^ ^34^, ^35^ ^]^ The MQW structure consists of repeated In_0.52_Al_0.48_As(barrier)/In_0.53_Ga_0.47_As(well) asymmetric double‐quantum‐well unit structures. The unit MQW structure was designed using a self‐consistent Poisson‐Schrödinger solver. The energy levels of the electron subbands and IST dipole matrix elements between two electron subbands were calculated using this solver. The MQW structure was optimized to obtain the largest IST dipole matrix elements at a wavelength of 6.25 µm. The layer sequence of the unit quantum well structure was 8.0/**5.0**/1.0/**1.7**/8.0 (in nm), where the boldface indicated In_0.53_Ga_0.47_As well layers, and the 5 nm‐thick well was n‐doped with doping density of 5.5 × 10^18^ cm^−3^. The unit quantum well structure was repeated seven times to form an MQW layer with a total thickness of 166 nm. An additional 10 nm‐thick In_0.53_Ga_0.47_As buffer layer and 5 nm‐thick In_0.53_Ga_0.47_As contact layer with a doping density of 5.5 × 10^18^ cm^−3^ were used on the top and bottom of the MQW layer (total thickness: 196 nm). The In_0.53_Ga_0.47_As contact layers acted as charge‐spreading layers for uniform potential distributions at the top and bottom metal contacts. The IST in the MQW structure can produce giant electro‐optic effects near the corresponding resonant frequency only for TM‐polarized light owing to the polarization formed along the surface‐normal direction (*z*‐direction here). The surface‐normal component of the dielectric function of the MQW layer can be expressed using the following equation:^[^ ^36^ ^]^

(1) $$\varepsilon_{z}\left(\omega \right)=\varepsilon_{\infty }+\frac{N_{e}{\left(ez_{12}\right)}^{2}}{\varepsilon_{0}\hbar \left(\left(\omega_{12}-\omega \right)-i\gamma_{12}\right)}$$

where *ε*_∞_ *, ε*_0_ *, e, N*_e_, ℏ, and *ω* represent the dielectric constant of the undoped semiconductor structure, vacuum permittivity, electron charge, average doping density in the MQW, the reduced Planck constant, and angular frequency of the incident light, respectively; *ez*_12_, *ω*_12_, and *γ*_12_ are the transition dipole moment element, IST frequency, and damping constant for the IST between electron sub‐bands 1 and 2, respectively. **Figure** **2**a,b shows the conduction band diagrams of one period of the MQW structure at the two different bias voltages of +1 and −1 V, respectively. When +1 V is applied over the 200 nm‐thick MQW layer (Figure 2a), the energy separation between the two electron subbands increases further from the 0 V case owing to the different Stark shifts of the spatially separated electron subbands. This results in a blue‐shifted IST wavelength. When −1 V is applied (Figure 2b), the IST energy decreases and the corresponding wavelength is red‐shifted. Figure 2c shows the calculated IST energies between the two electron subbands as a function of the applied electric field. The result exhibits almost linear IST energy (or wavelength) tuning from 188 meV (*λ* = 6.60 µm) to 208 meV (*λ* = 5.96 µm) for bias voltages ranging from −1 V (−50 kV cm^−1^) to +1 V (+50 kV cm^−1^) over the 200 nm‐thick MQW layer. The surface‐normal component of the dielectric constant for the designed MQWs structure was calculated by applying bias voltages from −1 to +1 V in steps of 0.2 V as shown in Figure S1 (Supporting Information).

**Figure 3:**
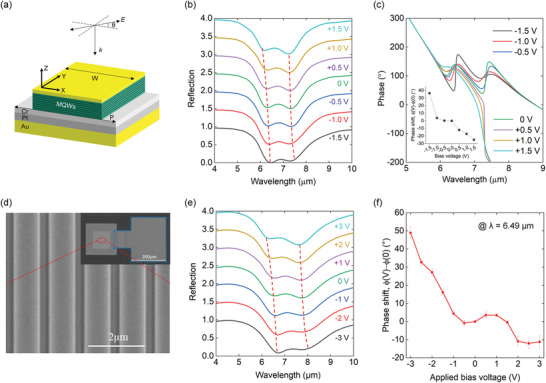
Electrical control of reflection spectra and phase. a) Schematic of the unit cell structure. The width (*W*) of the top plasmonic resonator together with the multiple quantum well (MQW) layer and the resonator array period (*P*) are 900 and 1300 nm, respectively. *E* represents the linearly polarized electric field and *k* represents ‐*z* directional incident wavevector. b,c) Simulation results for the b) reflection spectra and c) reflection phase spectra for different applied bias voltages from −1.5 to +1.5 V with a step of 0.5 V (inset: Calculated phase shift at 6.49 µm as a function of the applied bias voltage). d) Scanning electron microscopy (SEM) image of the fabricated metasurface. The blue dotted line (in the inset) indicates the top contact metal. e) Measured reflection spectra for different bias voltages from −3 to +3 V employing a step size of 1 V. For clarity, the simulated and measured reflection spectra at different bias voltages are offset from each other by 0.5. f) Phase shift measured by a Michelson‐type interferometer at 6.49 µm for applied bias voltages ranging from −3 to +3 V in steps of 0.5 V.

**Figure** **3**a shows the designed unit cell structure of the metasurface. The meta‐atom nanocavity consists of a 200 nm‐thick MQW layer sandwiched between a top Au layer and an optically thick Au back plane in a ridge resonator. The ridge resonators are designed to have a cavity resonance for *x*‐polarized incident light near the intersubband transition energy *E*_12_. The resonance generates enhanced *E*_z_ fields inside the MQW layer, which can be coupled to the IST. The coupled mode, called the intersubband polariton, can be tuned by applying a bias voltage through the metal resonator cavity, resulting in phase and amplitude modulation of the *x*‐polarized reflection beam. Figure 3b shows the calculated reflection spectra obtained by applying bias voltages from −1.5 to +1.5 V in steps of 0.5 V. We used a finite‐difference time‐domain (FDTD) solver (Lumerical) for the calculation. Figure 3b shows the reflection spectral splitting and anti‐crossing behavior, which indicates polaritonic coupling. The major spectral tuning occurred in the wavelength range between 6.04–7.44 µm, and the effect of reflection amplitude and phase tuning according to the bias voltage in this range was used. Figure 3c and inset of Figure 3c show the calculated reflection phase spectra of the *E*_x_ field and reflection phase shift at a wavelength of 6.49 µm, respectively, for different bias voltages from −1.5 to +1.5 V in steps of 0.5 V. A major phase shift occurred near the wavelengths of 6.45 and 7.3 µm. These are two asymptotic points where anti‐crossing occurs (cf. Figure 3b). For the calculation of reflection magnitude and phase spectra, we considered the effect of nonuniform conduction band bending caused by the formation of Schottky contacts at the MQW and Au interfaces (see Figure S2, Supporting Information). The physical parameters of the MQW used for the calculation are presented in Table S1 (Supporting Information). For an experimental demonstration, we fabricated metasurfaces comprising a two‐dimensional (100 × 100 µm) array of unit cells. The basic IST measurement results of our MQW structure and device fabrication processes are provided in Figures S3 and S4 (Supporting Information). Figure 3d shows a scanning electron microscopy (SEM) image of the fabricated metasurface, where the blue dotted line in the inset indicates the top contact metal for applying bias voltages to the MQW layer. Experimentally, the spectral splitting at 0 V confirmed the strong coupling between the plasmonic resonance and IST, while spectral tuning was observed by applying bias voltages from −3 to +3 V in steps of 1 V as shown in Figure 3e. The major spectral tuning occurs in the range between 6.51 and 7.72 µm. It is noted that the spectral peak position of the fabricated device was slightly red‐shifted and spectral broadening in the long‐wavelength region was observed, which is attributed to the influence of unwanted impurities during the sample fabrication process. As shown in Figure 3f, the reflection phase shift with respect to the 0 V phase at a wavelength of 6.49 µm for applied bias voltages from −3 to +3 V in steps of 0.5 V, was measured using a Michelson interferometer (see Figure S6, Supporting Information). During the phase‐shift measurement, the average and standard deviation of the phase shift were obtained through repeated measurements at five different positions of the interference pattern image. A maximum phase shift of 48.9° of the *x*‐polarized reflected light was obtained at an applied bias voltage of −3 V, and a total phase shift of over 60° was obtained in the applied voltage range. The experimental phase shift results show similar trends to the calculation results in the inset of Figure 3c, requiring higher bias voltages due to the effect of contact resistance at the MQW and Au interfaces. Increasing the bias voltage is expected to yield larger phase shifts, but the range between −3 and +3 V allows safe operation without damaging the device. The phase shift can be further increased through more uniform conduction band bending with formation of the ohmic contact and better quantum well structures, which can suppress the current level. The phase shift spectra were measured from 6.29–6.79 µm in steps of 0.1 µm at the fixed bias voltages of −3 and +3 V, and the experimental interference patterns for extracting the reflection phase are shown in Figures S6 and S7 (Supporting Information), respectively. Next, we demonstrate electrical tuning of the polarization state and electrical tuning of wavefront of the reflected beam, using the properties of electrically tunable reflection phase from the intersubband polaritonic metasurface.

**Figure 4:**
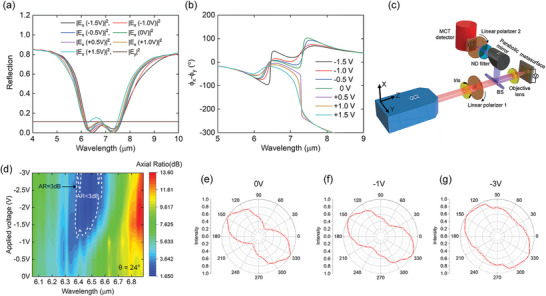
Electrically tunable quarter waveplate. a,b) Simulated power reflection spectra (a) and the simulated phase difference spectra between the reflection coefficients for *E*_x_ and *E*_y_ fields (b) for different bias voltages from −1.5 to +1.5 V in steps of 0.5 V (cf. Figure 3a). c) Optical setup for tunable beam polarization control (QCL: quantum cascade laser, BS: beam splitter, ND: neutral density, MCT: mercury cadmium telluride). d) Measured result of the axial ratio (AR) as a function of the applied bias voltage and wavelength for incident light polarized at *θ* = 24° to *x*‐axis. e–g) Polar plots of the measured intensity of the reflected beam analyzed by rotating the second linear polarizer in Figure 4c from 0° to 180° in steps of 10° for applied bias voltages of e) 0 V, f) −1 V, and g) −3 V at 6.49 µm wavelength. The intensities from 180° to 360° are replicated from the measured results from 0° to 180°.

To control the polarization state from the metasurface from linearly polarized incident light to circularly polarized reflected light (quarter waveplate operation), equal amplitudes and a phase difference of *π*/2 for the reflected *E*_y_ and *E*_x_ field components are necessary. The incident linear polarization was rotated with an azimuthal angle *θ*, as shown in Figure 3a, to ensure approximately equal amplitudes of the *E*_x_ and *E*_y_ fields. The incident *E*_x_ field component resonantly couples to the metasurface cavities and experiences the phase shift due to the cavity resonances. The incident *E*_y_ field does not resonantly couple to the metasurface cavities. Therefore, the amplitude and phase of the reflected *E*_y_ field did not change according to the bias voltage, and possessed constant values at each angle *θ*. The phase of the reflected *E*_x_ field was modulated by applying a bias voltage to produce a phase difference from the constant phase of the *E*_y_ field. The amplitude and phase response of the reflected *E*_x_ and *E*_y_ fields were analyzed for linearly polarized incident light at *θ* = 20° from the *x*‐axis. **Figure** **4**a,b shows the simulated reflection amplitude spectra of the *E*_x_ and *E*_y_ fields, and the phase difference spectra of *ϕ*_x_ − *ϕ*_y_, for different bias voltages, respectively. The reflection phase difference of about 50° is expected to occur near the wavelength of 6.5 µm when a negative bias voltage is applied (cf. Figure 4b). Thus, a right elliptically polarized reflection beam can be generated. For the experimental demonstration, the optical setup shown in Figure 4c was used. A wavelength‐tunable quantum cascade laser (QCL) with a wavelength tuning range of 6–7 µm was used as the linearly polarized light source. From the simulation results in Figure 4b, it is expected that larger phase tuning may be obtained in the wavelength range of 7.1–7.3 µm, but the proof‐of‐concept demonstration was conducted near the wavelength of 6.5 µm due to the narrow tunable phase spectral bandwidth near the wavelength of 7.2 µm and absence of a laser source in that wavelength range. The linear polarizer 1 for adjusting the incident polarization was set to 24° from the *y*‐axis, which was a slightly larger value compared to simulations because of the inevitable discrepancy between the metasurface absorption spectra in the simulation and experiment. Figure 4d shows the measured axial ratio (AR = 10log_10_(*I*_max_/*I*_min_), where *I*_max_ (*I*_min_) is the maximum (minimum) intensity of the reflected light) in the dB scale of the reflected light from the metasurface as a function of the wavelength and applied bias voltage. The AR was measured by rotating the linear polarizer 2 in front of the mercury cadmium telluride (MCT) detector from 0° to 180° in steps of 10°. Nearly circularly polarized reflected light with AR < 3 dB was obtained in the wavelength range of 6.37–6.57 µm for applied bias voltages from −0.5 to −3 V in steps of 0.5 V as shown in Figure 4d. We calculated the degree of circular polarization (DoCP) as shown in Figure S8 (Supporting Information). Over 95% of DoCP values were obtained in the wavelength range of 6.35–6.57 µm for an applied bias voltage of **–**3 V. This result is consistent with the region of AR < 3 dB. Figure 4e–g shows the polar plots of the reflected light intensity measured by rotating the second polarizer at 0, −1, and −3 V, respectively, at 6.49 µm. At 0 V, an AR of 6.85 dB was obtained, and by increasing the negative bias voltage, an AR of 1.94 dB was obtained at −3 V, which satisfied the condition of circularly polarized light. Thus, one can control the ellipticity of light according to the bias voltage and potentially use it as an electrically tunable quarter‐waveplate. Furthermore, analysis of the change in amplitudes of reflected E‐field was performed. As shown in Figure S9 (Supporting Information), the amplitude of *E*_x_ component (angle of 90°, 270° of the linear polarizer 2) was changed due to the strong coupling modulation but the amplitude of *E*_y_ component (angle of 180°, 360° of the linear polarizer 2) was not changed.

**Figure 5:**
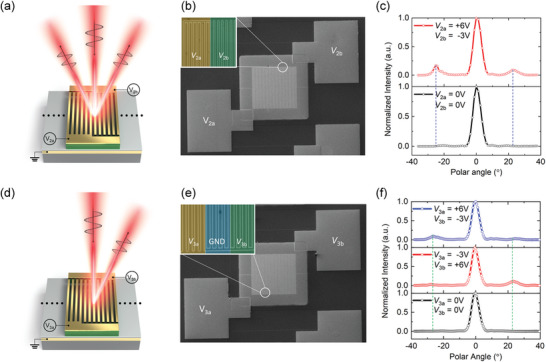
Dynamic beam manipulation. a,d) Schematics of a) electrically tunable phase grating metasurface and d) phase gradient metasurface. Scanning electron microscopy (SEM) images of the b) fabricated tunable phase grating metasurface and e) phase gradient metasurface. The insets are the magnified views of the contact formations. c) Measured far‐field profiles of the reflection beam intensity of the electrically tunable phase grating metasurface at two different bias conditions: *V*_2a_ = *V*_2b_ = 0 V (bottom panel) and *V*_2a_ = +6 V, *V*_2b_ = −3 V (top panel). f) Measured far‐field profiles of the reflection beam intensity of the electrically tunable phase gradient metasurface at three different bias conditions: *V*_3a_ = +6 V, *V*_3b_ = −3 V (top panel), *V*_3a_ = −3 V, *V*_3b_ = +6 V (middle panel), *V*_3a_ = *V*_3b_ = 0 V (bottom panel).

As a second application, electrically tunable phase‐grating metasurfaces and phase‐gradient metasurfaces were fabricated to experimentally demonstrate dynamic beam manipulations. For the two metasurfaces, groups of 12 ridge resonators in the *x*‐direction were used as supercells with a period *Γ*. Their operation is illustrated in **Figure** **5**a–d for electrically tunable beam diffraction and beam steering, respectively. The two metasurfaces were fabricated with a size of 200 × 200 µm by repeating the supercell 12 times. The SEM images of the processed metasurfaces are shown in Figure 5b–e. The phase‐gradient metasurface was fabricated with the same geometric parameters with a different bias contact formation (cf. Figure 5a,b). As shown in the inset of Figure 5b, the supercell of the phase‐grating metasurface is divided into two subsections with six ridge resonators, each connected to different bias contact pads *V*_2a_ and *V*_2b_. This effectively produces an electrically induced phase grating with a 50% duty cycle. The ± first order beam diffraction can be controlled by applying positive and negative bias voltages to *V*_2a_ (left contact pad) and *V*_2b_ (right contact pad), respectively. Figure 5c shows the experimental results of the electrically tunable beam diffraction as a function of the polar angle from −40° to +40° at an operating wavelength of 6.50 µm. For *V*_2a_ = *V*_2b_ = 0 V (bottom panel), only the zeroth order beam was observed. For *V*_2a_ = −3 V and *V*_2b_ = +6 V (top panel), the ± first‐order diffraction beams with an intensity of 15% compared to the zeroth order reflected beam intensity were observed at a diffraction angle of ±25°, exactly following the diffraction grating equation (*θ* = ± sin^−1^ (*mλ*/*Γ*)). For the beam diffraction efficiency calculation, we used the reflection efficiency of 4.68% at an operating wavelength of 6.50 µm when applying bias voltages of *V*_2a_ = *V*_2b_ = 0 V, as shown in Figure S10 (Supporting Information). When bias voltages *V*_2a_ = −3 V and *V*_2b_ = +6 V are applied, the efficiency of ± first‐order diffraction beam is 0.70% (the normalized beam diffraction efficiency is 15%), respectively. In the case of the rectangular phase grating with a 50% duty cycle, the zeroth order diffraction was completely suppressed when the phase difference was 180°.^[^ ^37^ ^]^ However, our phase‐grating metasurface exhibits an experimental phase difference of approximately 60° under the operating bias condition (cf. Figure 3f), resulting in a limited diffraction beam intensity. To verify the electrical dynamic modulation of the beam steering, we measured the positive angle beam steering signal from the metasurface by applying square voltage pulses of *V*_2b_ at 10 kHz in the range from −1.5 to +6 V, as shown in Figure S11 (Supporting Information). The output beam‐diffraction signal was modulated exactly following the applied bias voltage. Considering the device dimensions and *I*–*V* characteristics, the modulation speed mainly limited by the RC time constant of the metasurface was expected to be higher than 100 MHz. For the dynamic beam steering experiment, we used electrically tunable phase gradient metasurfaces, where the supercell was divided into three sections comprising four ridge resonators each. As shown in the inset of Figure 5e, a phase gradient was produced by applying no bias to the middle section of the supercell, positive voltage *V*_3a_ to the section of the supercell (left contact pad), and negative voltage to *V*_3b_ to the right section (right contact pad). Alternatively, we could exchange the values of the voltages applied to the left and right sections, respectively. Figure 5f shows the experimental results of selective beam‐steering measurements for two different DC bias voltages at normal incidence using an operating wavelength of 6.45 µm. The output signals were normalized to each zeroth intensity. For *V*_3a_ = +6 V (“2”) and *V*_3b_ = −3 V (“1”) as a phase sequence of “…210 210…”, the metasurface diffracted the beam only into the direction of the negative angles as shown in the top panel in Figure 5f. For the opposite bias condition with a phase sequence of “…01 2012…”, the diffracted beam was only presented in the direction of positive angles, as shown in the middle panel. The beam‐steering angle follows the generalized Snell's law, *θ* = sin^−1^ (*λ*/*Γ*). For beam steering efficiency calculation, we used the reflection efficiency of 4.24% at an operating wavelength of 6.45 µm when applying bias voltage *V*_3a_ = *V*_3b_ = 0 V as shown in Figure S10 (Supporting Information). When applying bias voltages *V*_3a_ = −3 V(+6 V) and *V*_3b_ = +6 V(−3 V) are applied, the efficiency of positive (negative) angle steered beam is 0.53% (0.45%). The normalized beam efficiency is 11.45% (9.63%) for positive (negative) angle steered beam. The performance comparison with other electrically tunable mid‐IR metasurface research results reported so far is summarized in Table S2 (Supporting Information).

## 3 Conclusion

In conclusion, we proposed and experimentally demonstrated electrically tunable intersubband polaritonic metasurfaces, in which the polaritonic spectral and phase were controlled by an external bias voltage through the QCSE associated with the IST in the MQW semiconductor material. Using an array of ridge metal‐MQW‐metal resonators, we experimentally demonstrated the multifunctional operation of our metasurface as an electrical polarization state control device and beam steering device. This advanced feature comes with the high modulation speed and can be utilized at a relatively low driving voltage. It is noted that reflection efficiency of the device may be improved using IST with narrower linewidth which can be obtained by using barrier modulation doping technique (see Figure S12, Supporting Information). The operating wavelength of the intersubband polaritonic metasurface can be easily adjusted over a wide range by controlling the quantum well structure, and extended to the near‐infrared region using other heterostructures such as AlGaN/GaN.^[^ ^38^ ^]^ The electrically tunable polaritonic metasurface may open new pathways for innovative applications, such as spatial light modulators, varifocal metalenses, and dynamic holography.

## 4 Experimental Section

### Numerical simulation

FDTD calculation and simulation were performed by using a commercial software (Ansys Lumerical 2021 R1). The optical properties for unit grating meta‐atom were calculated by applying boundary conditions with anti‐symmetric along the *x*‐direction, symmetric along the *y*‐direction, and perfectly matched layer (PML) along the *z*‐direction at normal incidence. The far‐field projection from unit supercell was applied with periodic boundary conditions along the *x*, *y*‐directions, and PML along the *z*‐direction. The reflection spectra and phase responses, under incident *x*‐polarized plane wave, were extracted from S‐parameter Analysis Group, and far‐field projection data was extracted from the 2D Frequency‐domain field and power monitor. The unit grating meta‐atom and unit supercell were simulated with global mesh level 4, and up to 1  × 10^−5^ of auto shutoff min to optimize run time, collected data, and precise calculations. The Drude parameters of gold was used as “Au (Gold) – Palik” in an embedded material group.

### Device fabrication

Device fabrication process is shown in Figure S4a (Supporting Information). A 300 nm‐thick In_0.53_Ga_0.47_As layer (first etch‐stop layer), a 100 nm‐thick InP layer (second etch‐stop layer), and 200 nm‐thick MQW layer are epitaxially grown on a InP substrate. The InP wafer with MQW layer and a Si wafer were flip‐wafer bonded to each other with thermo‐pressure after metal deposition in the order of 20 nm‐thick Cr, 50 nm‐thick Pt, and 150 nm‐thick Au, respectively. The Cr and Pt were used for a good adhesion layer and Au thermal diffusion blocking layer during the flip wafer bonding process, respectively. The InP substrate on the MQW side was removed by hydrochloric acid diluted with deionized water (HCl:DI = 300 mL:100 mL) after mechanical polishing of the InP substrate thinned down to 140 µm thickness. Subsequently, the etch stop layers, as the 300 nm‐thick InGaAs and 100 nm‐thick InP layers, were selectively wet‐etched by using phosphoric acid diluted with hydrogen peroxide and deionized water (H_3_PO_4_:H_2_O_2_:DI = 10 mL:10 mL:380 mL) for InGaAs layer, and hydrochloric acid for InP layer, respectively. A 6 nm‐thick Cr layer and a 60 nm‐thick Au layer were deposited on the MQW surface by electron‐beam evaporator. A 400 nm‐thick Si_x_N_y_ layer as dry etch mask layer was deposited on the Au layer deposited by using a plasma enhanced chemical vapor deposition (PECVD) (SiH_4_: 220 sccm, NH_3_: 50 sccm, Pressure: 1.5 Torr, Temperature: 200 °C), and then the one‐dimensional grating array with the size of 200 × 200 µm were patterned by an electron beam lithography with electron‐beam resist of ARP‐6200 (Allresist Inc.). After dry‐etching the Si_x_N_y_ and the top Au layer with the e‐beam resist patterned mask by using inductively coupled plasma (ICP) (CF_4_: 20 sccm, CHF_3_: 30 sccm, Pressure: 4 mTorr, AC power: 1000 W, DC power: 150 W), the MQW layers were dry‐etched (Cl_2_: 45 sccm, N_2_: 15 sccm, Pressure: 4 mTorr, AC power: 2000 W, DC power: 300 W) with the patterned Si_x_N_y_ mask layer. After mesa patterning with a size of 400 × 400 µm with a photoresist (PR) mask (AZ5214E, MicroChem.), the background Au and MQW layers were removed using ICP dry etching and wet chemical etching, respectively. Then, a 400 nm‐thick Si_x_N_y_, as a dielectric spacer layer between the mesa patterned Au and bottom metal layers, was deposited again using the PECVD, and the grating array and common bottom contact were opened by using the buffered oxide etchant (BOE) solution after photo‐patterning. The top contact pad was deposited in the order of 20 nm‐thick Cr, 300 nm‐thick Cu, 10 nm‐thick Cr and 50 nm‐thick Au layers, and then lift‐off process.

## Conflict of Interest

The authors declare no conflict of interest.

## Supporting information

Supporting Information

## Data Availability

The data that support the findings of this study are available from the corresponding author upon reasonable request.
