# Supplementary material for: Electrical Phase Modulation Based on Mid‐Infrared Intersubband Polaritonic Metasurfaces
Source: Adv Sci (Weinh). 2023 Apr 7;10(16):2207520. doi: 10.1002/advs.202207520 (PMC10238174; doi:10.1002/advs.202207520)
Supplement: Supplementary file 1 — Supporting Information [file ADVS-10-2207520-s001.pdf]

ADVANCED SCIENCE
Open Access

Supporting Information

for *Adv. Sci.*, DOI 10.1002/advs.202207520

Electrical Phase Modulation Based on Mid-Infrared Intersubband Polaritonic Metasurfaces

*Hyeongju Chung, Inyong Hwang, Jaeyeon Yu, Gerhard Boehm, Mikhail A. Belkin and Jongwon Lee**

Supporting Information

Electrical phase modulation based on mid-infrared intersubband polaritonic metasurfaces

Hyeongju Chung[1‡], Inyong Hwang[1‡], Jaeyeon Yu[1], Gerhard Boehm[2], Mikhail A. Belkin[2], and Jongwon Lee[1]*

H. Chung, I. Hwang, J. Yu, Prof. J. Lee

[1] Department of Electrical Engineering, Ulsan National Institute of Science and Technology (UNIST), Ulsan 44919, Republic of Korea

E-mail: jongwonlee@unist.ac.kr

G. Boehm, Prof. M. A. Belkin

[2] Walter Schottky Institute, Technical University of Munich, Garching 85748, Germany

Keywords: metasurface, phase modulation, multiple quantum well, intersubband transitions, mid-infrared

Calculation of dielectric constant of designed MQW structure.

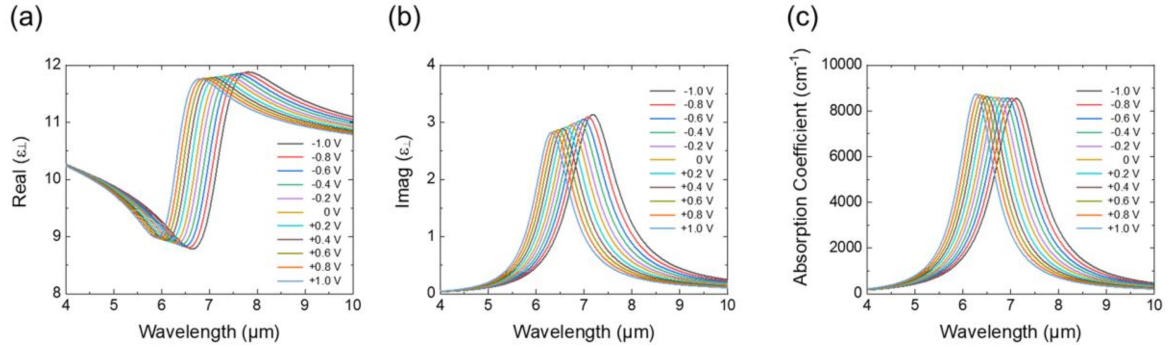

Figure S1. The real (a) and imaginary (b) parts of surface normal component of dielectric constant ($\varepsilon_{\perp}$) and absorption coefficient (c) for different bias voltages from −1 V to +1 V with 0.2 V step. The dielectric constant was calculated using the equation (1) in the main manuscript with physical parameters obtained from the self-consistent Poisson-Schrodinger solver. The absorption coefficient of the MQW structure was calculated from the relation with the imaginary part of dielectric constant as $\alpha = \frac{4\pi}{\lambda}\mathrm{Im}\left(\sqrt{\varepsilon_{\perp}}\right)$.

Non-uniform band bending and physical parameters of MQW structure.

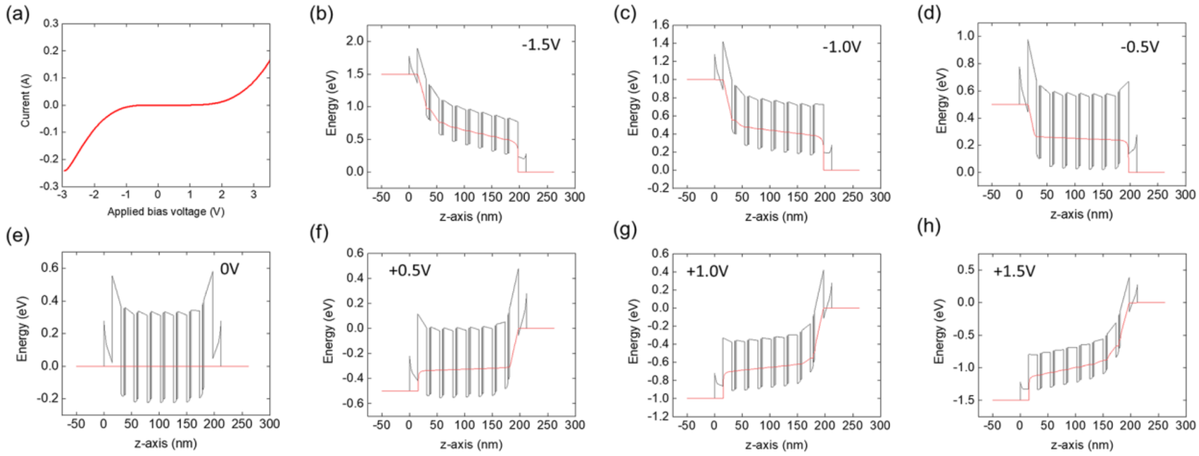

Figure S2. (a) I-V characteristic of the fabricated metasurface. The experimental I-V curve indicates the formation of Schottky contacts at the top and bottom MQW-metal interfaces. (b-h) Simulation results of conduction band bending for applied bias voltages from −1.5 V to +1.5 V with 0.5 V steps, assuming Schottky contact formation at the top and bottom surface of the MQW layer.

(a)

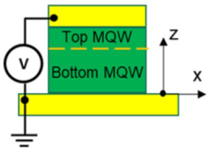

Applied bias voltage (V)	Physical parameters in bottom MQW layer region (0 < z < 176 nm)			Physical parameters in top MQW layer region (176 < z < 200 nm)		
	Electric field (kV/cm)	E_{12} (meV)	Z_{12} (nm)	Electric field (kV/cm)	E_{12} (meV)	Z_{12} (nm)
-1.5	28	177.8895	1.62603	177	144.6483	1.82885
-1.0	10	182.2922	1.60844	122	156.3281	1.74158
-0.5	2	183.9592	1.60261	42	174.6189	1.6402
0	0	184.4248	1.60096	0	184.4248	1.60096

(b)

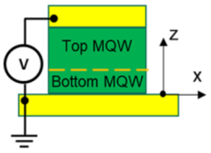

Applied bias voltage (V)	Physical parameters in bottom MQW layer region (0 < z < 24 nm)			Physical parameters in top MQW layer region (24 < z < 200 nm)		
	Electric field (kV/cm)	E_{12} (meV)	Z_{12} (nm)	Electric field (kV/cm)	E_{12} (meV)	Z_{12} (nm)
+0.5	-63	199.1781	1.558473	-2	185.2842	1.596374
+1.0	-156	218.1967	1.532214	-8	186.6764	1.591791
+1.5	-207	226.7047	1.528421	-27	191.0565	1.578541

Table S1. The extracted physical parameters of MQW structure using Poisson-Schrodinger solver (Nextnano) (a) for 0V and negative bias voltage and (b) positive bias voltage. For the negative bias voltages, the physical parameters of the MQW layer were extracted at bottom ($0 \leq z \leq 176$ nm) and top ($176 \leq z \leq 200$ nm) region separately considering non-uniform band bending of the MQW layer (cf. Figure S2). For the positive bias voltages, the physical parameters of the MQW layer were extracted at bottom ($0 \leq z \leq 24$ nm) and top ($24 \leq z \leq 200$ nm) region separately. In the table, E_{12} and Z_{12} are the intersubband transition energy and transition dipole element between electron sub-bands 1 and 2.

Intersubband absorption measurement and parameter extraction.

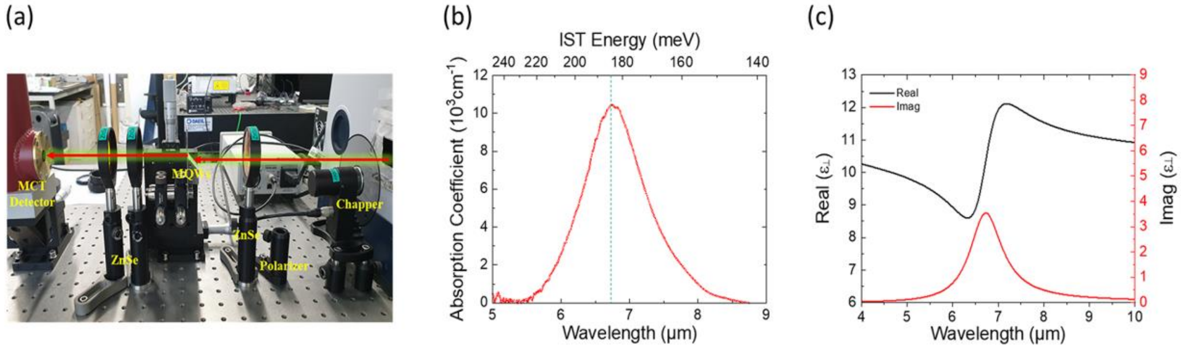

Figure S3. (a) Optical setup for the intersubband absorption measurement. Mid infrared light with TM polarization (signal) and TE polarization (background) passing through the MQW piece polished both sides with a slope of 45° were measured by the MCT detector. (b) Measured intersubband absorption coefficient spectrum at 0V. The absorption coefficient was converted from the measured absorbance spectrum considering the effective path length inside of the MQW sample. (c) Calculation result of surface normal component of real (black) and imaginary (red) part of the dielectric constant of the MQW structure.

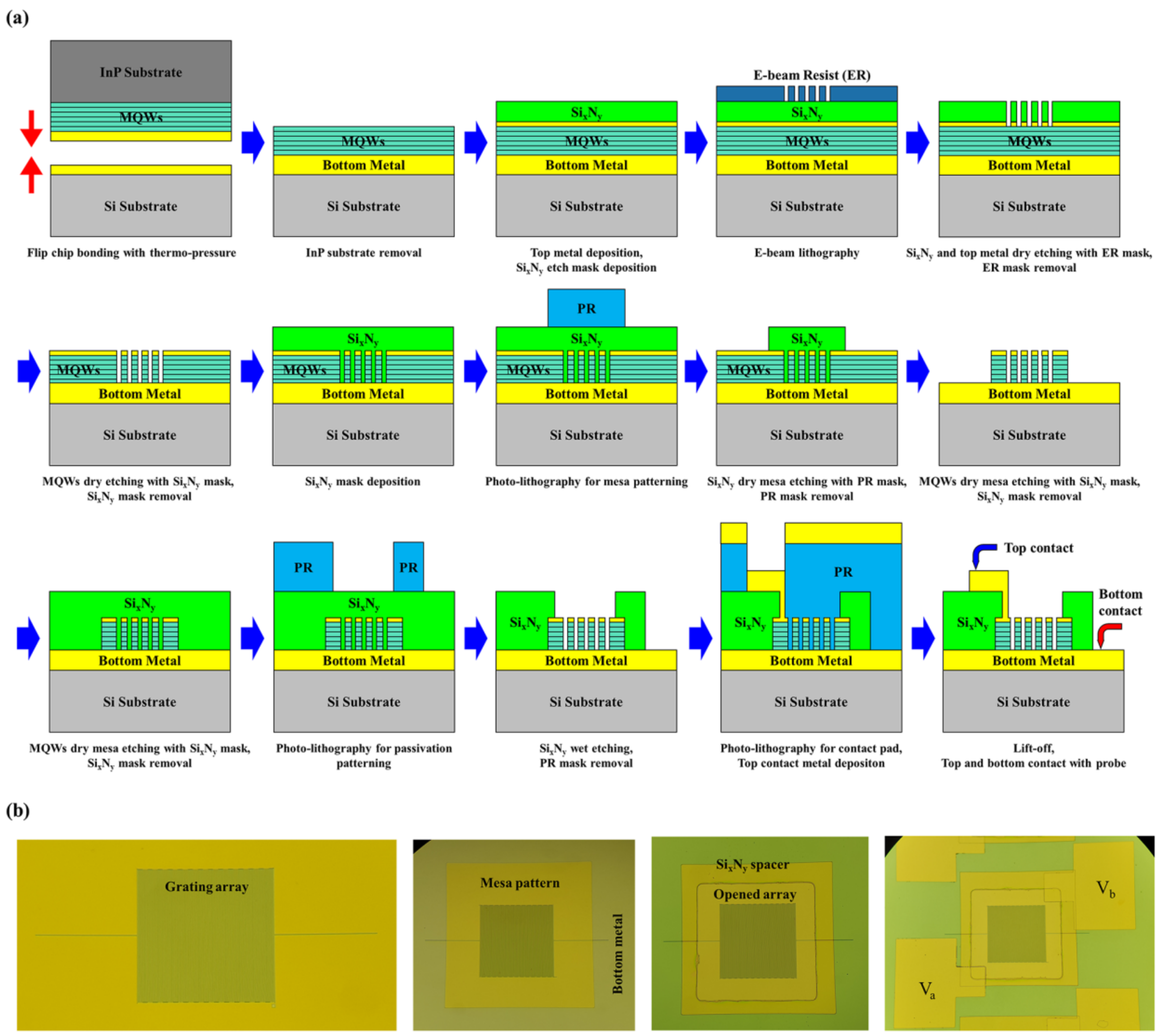

Figure S4. (a) Device fabrication processes. (b) optical microscope images of the fabricated metasurface. From the left, one-dimensional grating array, mesa-etched pattern, Si_xN_y spacer with array opening and final device with top contact pads.

The calculated result of modulation depth

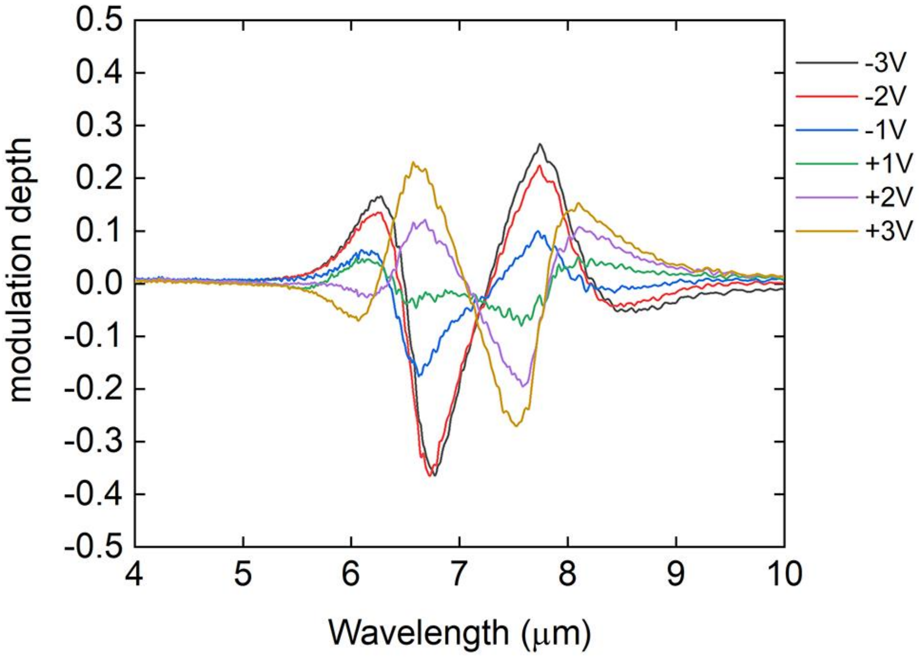

Figure S5. the calculated result of modulation depth from the reflected spectrum in Figure 3e. the modulation depth is defined as $\frac{A_v - A_{0V}}{A_v + A_{0V}}$, where A_v is refleced spectrum for applied bias voltage v and A_{0V} is reflected spectrum for applied bias voltage 0V. the modulation range can be identified very broad from 5.2μm to 9.5μm wavelength and especially, the modulation depth can be reached to the -36% for applied bias voltage -3V at 6.77μm wavelength.

Phase measurement setup and measured phase shift.

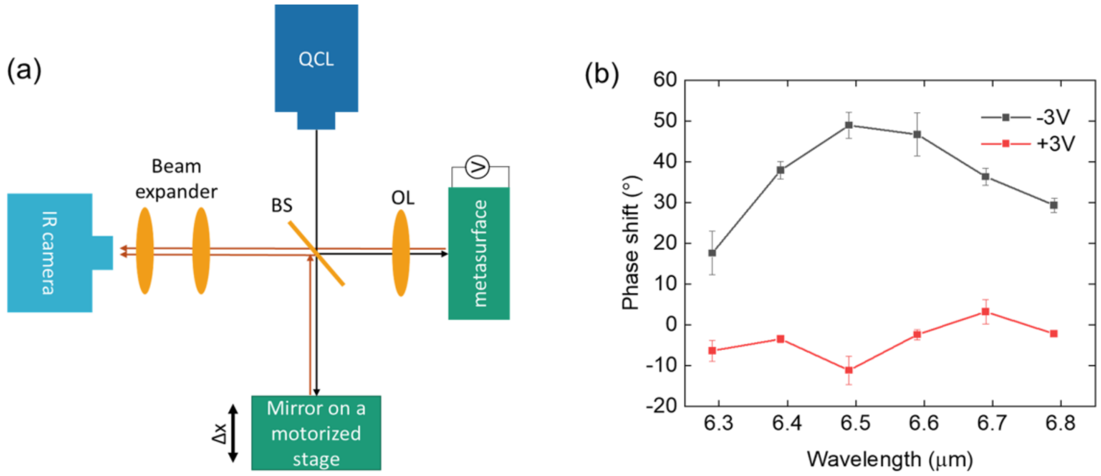

Figure S6. Phase measurement setup and phase shift result according to wavelength. (a) Meausrement setup of Michelson-type interferometer (BS:beam splitter, OL: Objective lens.). Infrared light from quantum cascade laser (QCL) is incident onto the BS and split into the metasurface and a mirror mounted on a motorized stage. Reflected lights from the metasurface and the mirror are incident to the BS and beam expender. Interference fringe pattern is formed and captured with infrared camera (pyrocam Ⅳ). (b) Measured phase shift according to the wavelength and applied bias voltages of −3V and +3V to the metasurface. Red and black square dot and error bar means the average and standard deviation value of five different positions in the metasurface region.

Interference patterns and phase extraction.

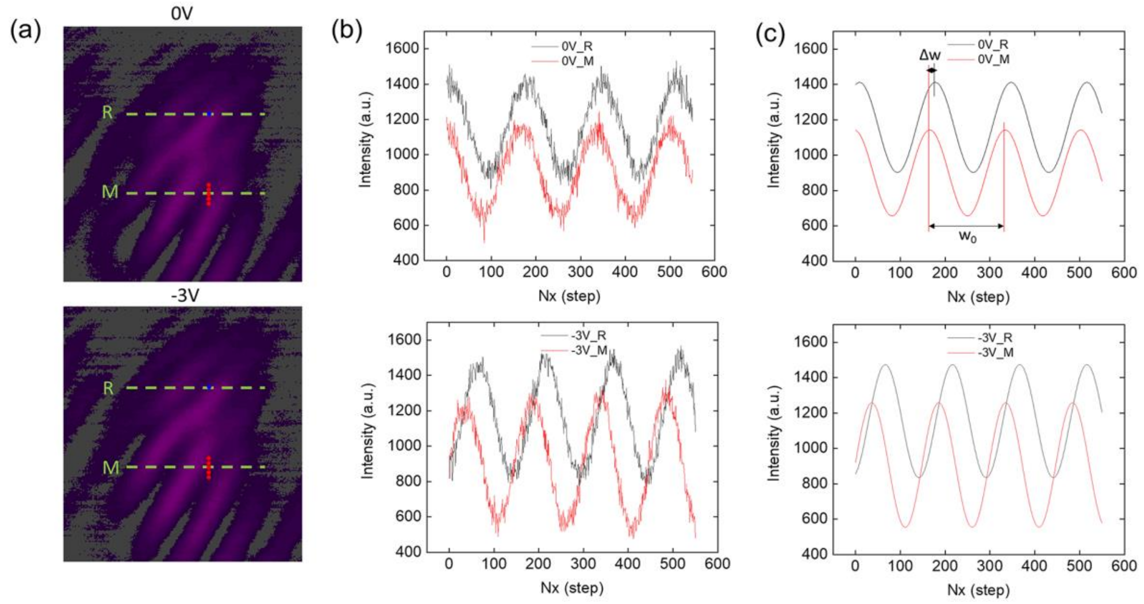

Figure S7. Phase measurement of intersubband polaritonic metasurface. (a) Captured image of the interference fringe pattern at 0V (top) and -3V (bottom), respectively, when light of 6.49μm wavelength was incident to the boundary line between mirror and metasurface. 'R(reference)' means the mirror of the metasurface and 'M(metasurface)' means the array pattern of the metausrfrace. The blue dot indicates reference point in the mirror region outside of metasurface and the five red dots indicate the different measured positions to extract phase shift in the array region of the metasurface. (b) Measured intensity of one position of interference fringe pattern by applying bias voltages to the metasurface at 0V(top) and −3V(bottom)(λ=6.9μm). (c) Fitted intensity of one position of interference fringe pattern of the results in (b). By moving (Nx step ≈ 40nm) the mirror on a motorized stage as shown in Figure S6(a), we used the formula $2\pi\Delta w/w_0$ to calculate the phase shifts, where Δw is the shift step numbers of the minimum (or maximum) intensity of the metasurface applied with −3V compared to minimum (or maximum) intensity of the metasurface applied with 0V and w_0 is the step numbers of one period between adjacent minimum (or maximum) intensities when applied with 0V.

The calculated result of Degree of circular polarization

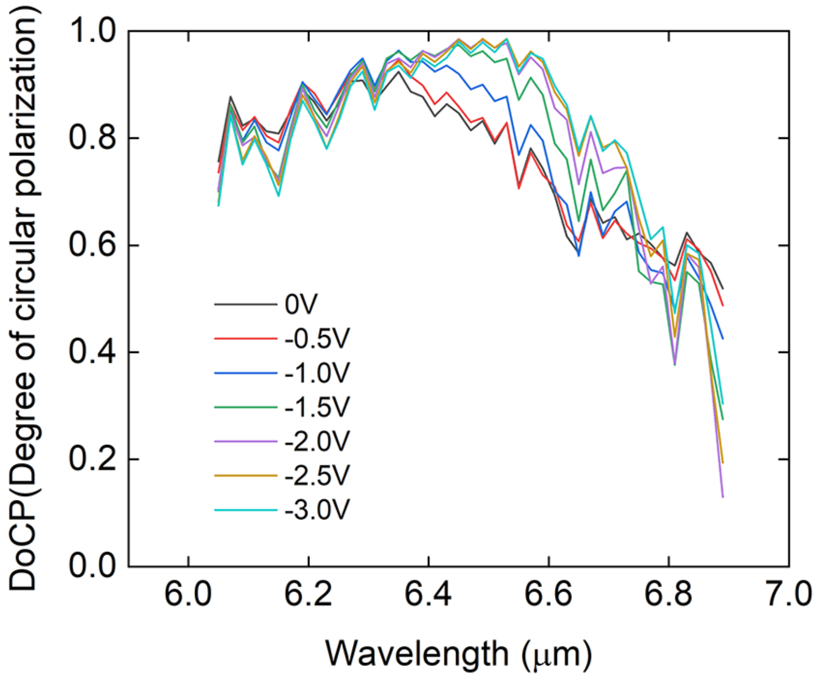

Figure S8. The calculated result of degree of circular polarization (DoCP).

For DoCP of waveplate metasurface, the Stokes parameters can be defined as below:

$S_0 = I_x + I_y$, $S_1 = I_x - I_y$, $S_2 = I_{+45^\circ} - I_{+135^\circ}$, $S_3 = I_{RCP} - I_{LCP}$, where I_x, I_y, I_{+45°, I_{+135° is the intensity of x, y, 45°, 135° linear polarized light, respectively and I_{RCP} and I_{LCP} is the intensify of right and left circular polarized light, respectively. We assumed that $S_0^2 = S_1^2 + S_2^2 + S_3^2$ because the quantum cascade laser has high coherence. S_1 and S_2 can be calculated and the S_3 can be calculated by $\sqrt{S_0^2 - S_1^2 - S_2^2}$. The DoCP is defined by $|S_3|/|S_0|$. As shown in Figure S8, The DoCP values are over 95% from 6.35μm to 6.57μm wavelength when applied bias voltage is -3V. This analysis is consistent with the AR (Axial Ratio) < 3dB region (ranged from 6.37 to 6.57 wavelength) result of Figure 4d when applied bias voltage is -3V.

The analysis of amplitude of reflected E-field components for polarization control

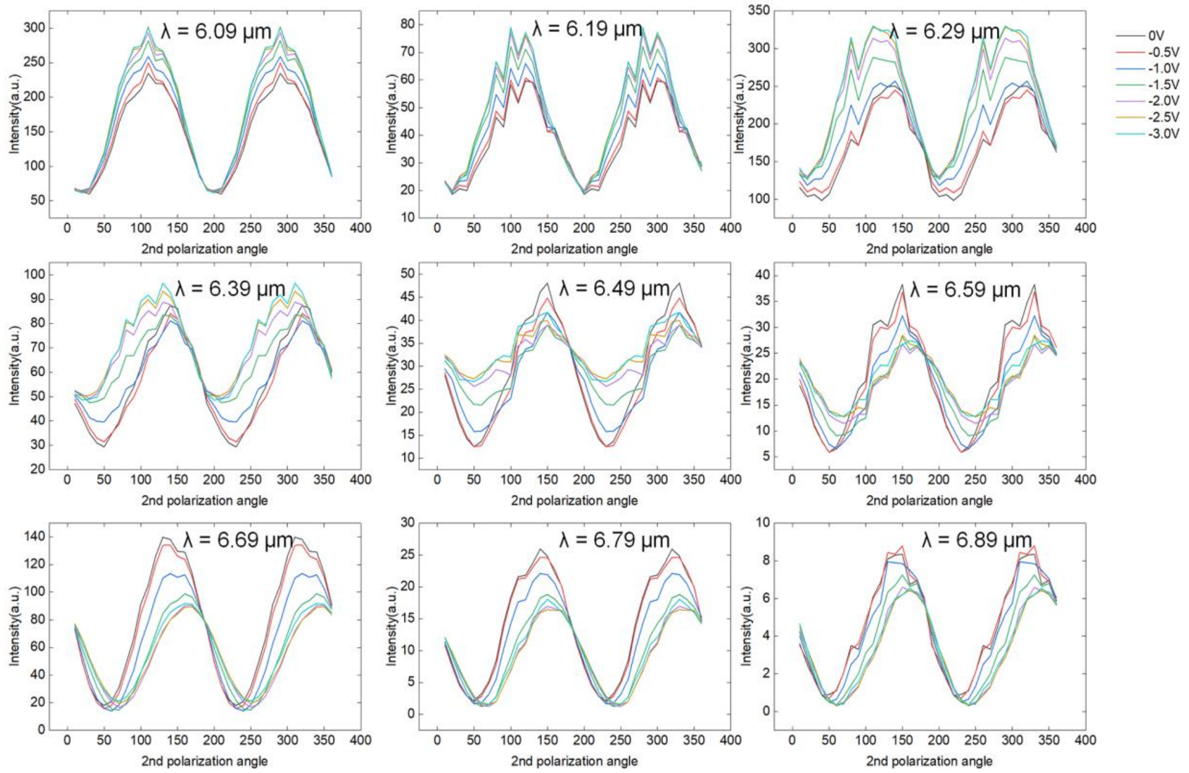

Figure S9. The amplitude of reflected E-field shown in Figure 4 by rotating linear polarizer 2 with 10° step. 90° (270°) polarization angle represents amplitude of E_x-component that was changed the amplitude according to applying bias voltage and 180° (360°) polarization angle represents amplitude of E_y-component that was not changed the amplitude according to applying bias voltage.

Reflection spectrum of beam manipulation metasurface

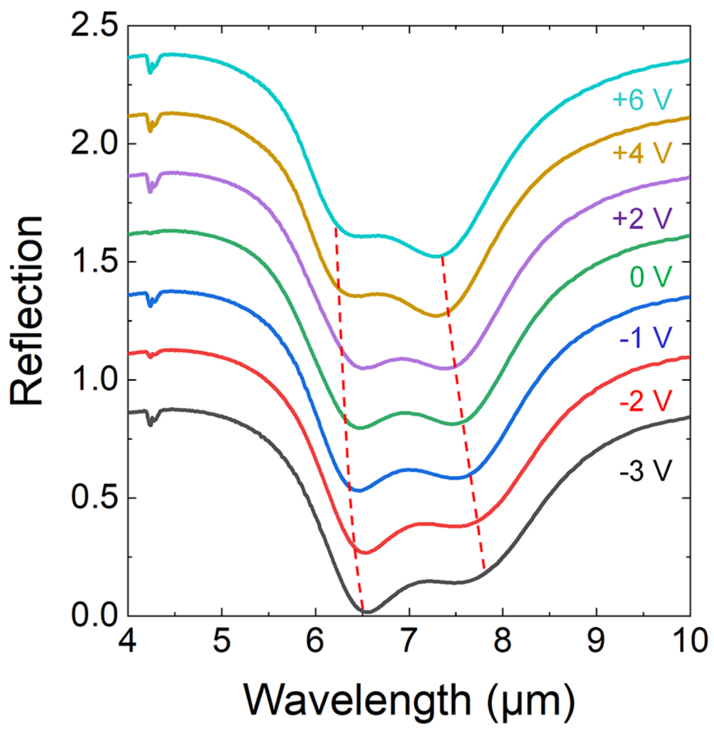

Figure S10. Reflection spetra of beam manipulation metasurface by applying bias voltages from -3V to +6V. For beam diffraction and beam steering when 0V bias applied, the reflection efficiency is 4.68%(λ=6.50μm) and 4.24%(λ=6.45μm), respectively.

Dynamic modulation of the reflection beam from the metasurface.

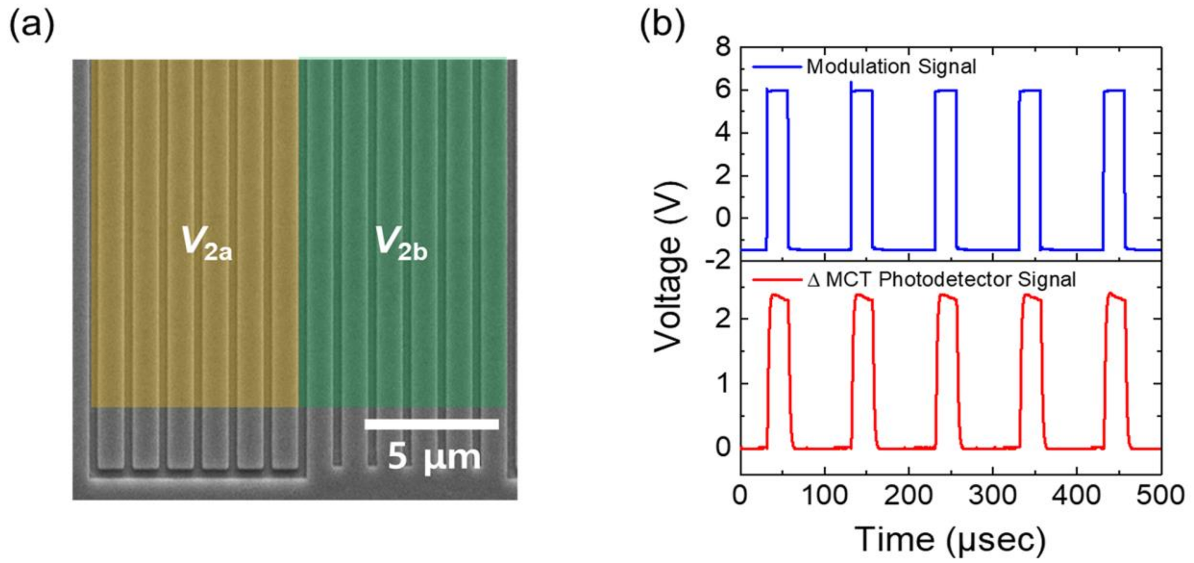

Figure S11. (a) SEM image of electrically tunable phase grating metasurface with a phase sequence of '…010…', where the $V_{2a} > 0$ V (yellow region) and $V_{2b} < 0$ (green region) are phase of '0' and '1', respectively. Dimensions of the metasurfaces are Γ_s = 16.44 μm, P (period) = 1.37 μm, L (line width) = 0.98 μm. (b) The applied square voltage pulse of V_{2b} with 10 kHz in range from −1.5 V to +6 V (top panel) and the MCT photodetector signal difference of +1st order diffraction intensity from the phase grating metasurface (bottom panel).

Effect of IST linewidth on the amplitude and phase tuning

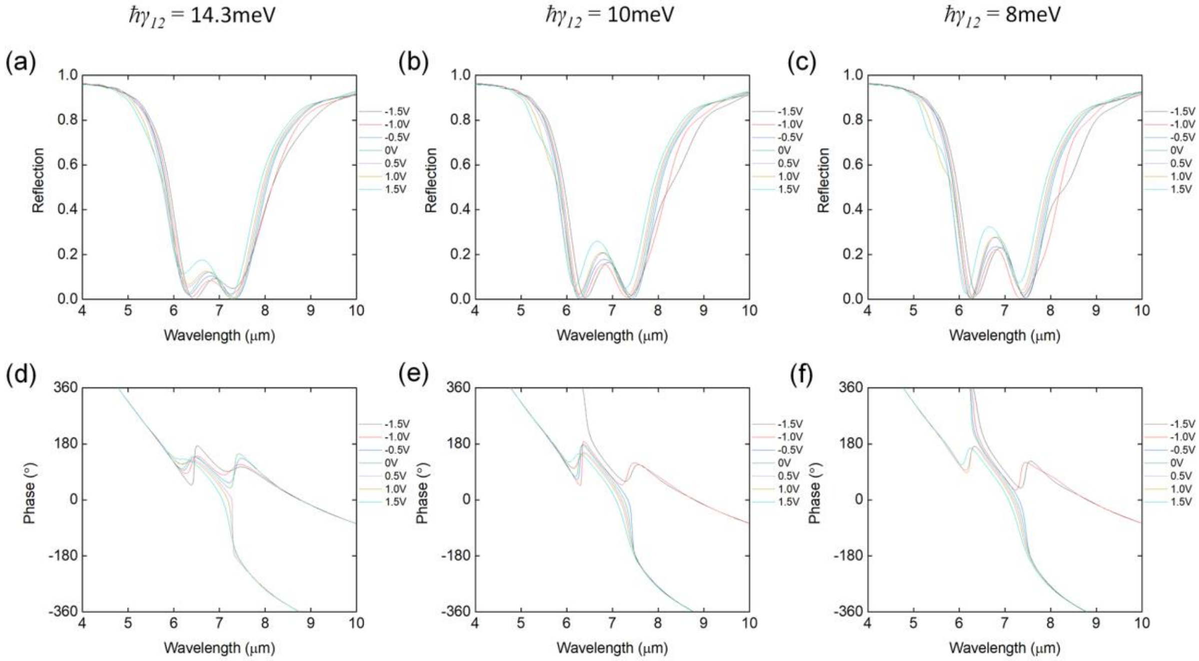

Figure S12. The calculation results of reflection (a,b,c) and phase (d,e,f) spectra according to the $\hbar\gamma_{12}$ =14.3, 10 and 8 meV, respectively.

Ref.	Operating wavelength	Used material	Modulated phase	Polarization control	Wavefront control
[1]	5.94μm	Indium Tin Oxide (ITO)	~180°	N/A	N/A
[2]	8.5μm	Graphene	~237°	N/A	N/A
[3]	3~5μm	$Ge_2Sb_2Te_5$	N/A (only AM)	N/A	N/A
[4]	2.5~4.5μm	VO_2	N/A (only AM)	N/A	N/A
This work	6.49μm	InGaAs/InAlAs	~60°	Linear to circular polarization	Beam diffraction modulation (0.7%)/ Beam steering (0.53%)

Table S2. Comparison of properties of electrically tunable mid-infrared metasurfaces for amplitude or phase modulation between our work and other related metasurfaces. (AM: amplitude modulation)

References

[1] J. Park, J.-H. Kang, S. J. Kim, X. Liu, M. L. Brongersma, *Nano letters* **2017**, 17, 407.

[2] M. C. Sherrott, P. W. Hon, K. T. Fountaine, J. C. Garcia, S. M. Ponti, V. W. Brar, L. A. Sweatlock, H. A. Atwater, *Nano letters* **2017**, 17, 3027.

[3] W. Dong, Y. Qiu, X. Zhou, A. Banas, K. Banas, M. B. Breese, T. Cao, R. E. Simpson, *Advanced Optical Materials* **2018**, 6, 1701346.

[4] L. Liu, L. Kang, T. S. Mayer, D. H. Werner, *Nature communications* **2016**, 7, 13236.
